# A Colorimetric Approach to Identifying Single-Nucleotide Mutations Using Gold-Based Magnetic Nanoparticles

**DOI:** 10.3390/ijms27198809

**Published:** 2026-10-01

**Authors:** Anna Epanchintseva, Julia Shyiskaya, Julia Poletaeva, Ekaterina Baeva, Evgenia Kozyreva, Evgeniya Dyudeyeva, Mariya Khokhlova, Inna Pyshnaya

**Affiliations:** 1 Knorre Institute of Chemical Biology and Fundamental Medicine, Siberian Branch of the Russian Academy of Sciences, Novosibirsk 630090, Russia; y.shuiskaya@alumni.nsu.ru (J.S.); gorbunova-ekaterina@inbox.ru (E.B.); malova.ev.an@gmail.com (E.K.); jenyadudeeva@gmail.com (E.D.); 2 Institute of Solid State Chemistry and Mechanochemistry, Siberian Branch of the Russian Academy of Sciences, Novosibirsk 630090, Russia; mariakhokhlova21@gmail.com

**Keywords:** magnetic hybrid nanoparticles, Fe_3_O_4_@Au, core–satellite, magnetite, gold nanoparticles, drug-resistant forms of tuberculosis, single-nucleotide polymorphism, fluoroquinolones, tandem duplexes, noncovalent adsorption, colorimetric detection

## Abstract

Detection of single-nucleotide mutations (SNPs) in the genome of infectious agents is one of the ways to identify their drug-resistant strains. Antibiotic-resistant strains of *Mycobacterium tuberculosis* (MTB) impede advances in the fight against the spread of tuberculosis. Diagnostic tools exist for both MTB itself and its drug-resistant strains, most of which are technically complex, resource-intensive, and time-consuming. This paper presents the development of a colorimetric test system, requiring no expensive equipment, for the rapid and accurate determination of the SNPs associated with drug-resistant forms of MTB to fluoroquinolones in modeled DNA targets (198 bp). For this purpose, nanoconstructs based on gold nanoparticles (AuNPs) and hybrid magnetic Fe_3_O_4_@Au NPs were created, leveraging the advantages of each nanomaterial. The use of AuNPs provides a basis for colorimetric detection due to their high optical absorption. Fe_3_O_4_@Au NPs enable samples to respond to an external magnetic field. The study demonstrates the ability to reliably discriminate between wild-type and mutant-model DNA targets in the nanomolar concentration range within 15 min. Visual analysis and optical absorption spectroscopy were used to evaluate the detection results. The application of an external magnetic field allows for enhanced colorimetric distinction between wild-type target samples and those containing an SNP.

## 1. Introduction

The spread of drug resistance to antibiotics in bacterial agents, particularly in *Mycobacterium tuberculosis* (*M. tuberculosis*, MTB), threatens advances in the fight against the global epidemic of tuberculosis. The treatment cycle for tuberculosis and multidrug-resistant tuberculosis (MDR-TB) is lengthy and costly [1,2]. Therapy for patients with first-line drugs (rifampicin and isoniazid) often leads to drug resistance in MTB; the subsequent use of second-line drugs (fluoroquinolones (levofloxacin, moxifloxacin), rifapentine, pyrazinamide, etc.) [3,4] leads to pre-extensively drug-resistant tuberculosis (pre-XDR-TB) [5], which is associated with an unfavorable treatment prognosis [6].

To identify drug-resistant MTB strains [7,8,9,10,11,12,13], culture-based and genotypic methods are used. However, the WHO currently recommends the use of multiplex DNA-amplification systems, such as the PCR-based Xpert MTB/RIF assay, which enable the simultaneous diagnosis of both pathogens and their drug resistance to rifampicin and other antibiotics [9]. These methods, despite their reliability and low detection limit, require laboratory equipment with costly instrumentation and highly qualified personnel, i.e., they are resource- and time-consuming. Clearly, there is a high demand for the development of structurally simple, rapid and sensitive test systems for the qualitative detection of MDR-TB [10].

Such systems include the detection of nucleic acids (NA targets) through their interaction with an oligonucleotide probe. Numerous variants of electrochemical, optical, photothermal, and fluorescence quenching-based sensors have been published [14,15,16,17,18,19,20,21,22,23,24,25]. Gold nanoparticles (AuNPs), magnetic nanoparticles (MNPs) of iron oxide, quantum dots, carbon-based materials, as well as hybrid NPs are typically used as carriers in these test systems. AuNP-based colorimetric sensors for the detection of the MTB IS6110 gene are characterized by a limit of detection (LOD) of 0.02 nM and a detection time of approximately 60 min [21]. Photothermal sensors allow us to distinguish the presence or absence of MTB DNA with an LOD of 2.8 × 10^−4^–3.9 × 10^−2^ µM and an analysis time of 40–120 min [22,23]. Electrochemical sensors can detect the picomolar and attomolar levels of DNA targets [17,25] and are being successfully applied in detecting MTB-associated SNPs [16]. However, these methods are associated with limitations regarding the use of expensive or rare equipment and complex sample preparation. Colorimetric sensors often do not require expensive equipment. For instance, in [21], the paper substrate of the sensor is printed using a standard printer, and wax is used to create distinct zones for samples. However, the high production cost of AuNPs increases the overall cost of AuNP-based assays. It should be noted that only a limited number of studies present the development of nonamplification test systems for the detection of single-nucleotide mismatches in the NAs that lead to MTB drug resistance. These are predominantly electrochemical sensors [16], specifically for the detection of MTB resistance to rifampicin in the *rpoB* gene [19], and fluorescence quenching-based sensors using cobalt-containing quantum dots for the detection of mutations in the *rpoB* gene and in the *katG* gene associated with isoniazid resistance [20].

Hybrid NPs—namely nanoparticles based on gold and iron oxide (MAuNP or Fe_3_O_4_@Au)—are promising variants of MNPs due to their combination of the advantageous properties of both components in a single object. AuNPs are attractive due to their intense color and easily detectable shift in the optical absorption maximum upon aggregation. The phenomenon of surface plasmon resonance on the gold coating surface expands the possibilities of applying MAuNP NPs in biosensors [26,27]. Moreover, the simplicity of synthesis, the biocompatibility of AuNPs, and their ability to effectively bind NAs and proteins are their advantages for practical application [14]. MNPs are used in many biomedical applications. Their key property is their susceptibility to external magnetic fields. In diagnostic systems, MNPs can be used (1) for the enrichment and separation of samples based on their magnetic properties, (2) for signal enhancement, and (3) for signal detection based on their magnetic, optical, or electrical properties [28,29]. Iron oxide nanoparticles with magnetite (Fe_3_O_4_) or maghemite (γ-Fe_2_O_3_) structures are the most widely used MNPs in biomedical applications due to their ease of synthesis, low cost, and high biocompatibility [30].

The present work focuses on the construction of nanostructures composed of AuNPs and hybrid magnetic Fe_3_O_4_@Au nanoparticles (MAuNPs). The combination of the physicochemical properties of these nanomaterials is aimed at the colorimetric detection of single-nucleotide substitutions (SNPs) that determine MTB resistance to fluoroquinolone antibiotics, within model oligonucleotide sequences using specific probes. The probes were efficiently noncovalently adsorbed onto AuNPs and MAuNPs through their functionalization with hydrophobic groups or conjugation with BSA. The high optical density of AuNPs in the visible spectral range serves as the physical basis for recording the analytical signal. Magnetic NPs enable the application of magnetic separation technology to enhance the analytical signal. The study demonstrates the discrimination of wild-type and mutant DNA targets in the nanomolar concentration range. The verification of the detection results was carried out by visual inspection and optical spectroscopy. It was established that the application of an external magnetic field provides a reliable increase in colorimetric differences between samples of the two target types. The novelty of the proposed study lies in the simultaneous use of AuNPs, MAuNPs, and DNA probes with the magnetic enrichment of samples for signal amplification. Solution-based colorimetric test systems like the one proposed here require minimal equipment and materials, namely standard test tubes and a laboratory thermostat. Using alternative magnetic hybrid NPs reduces the cost of developing the test system. The detection of SNPs in microbial genomes is widely used to identify drug-resistant strains. The versatility of the proposed approach lies in its potential application to targets associated with other infectious agents.

## 2. Results and Discussion

### 2.1. Synthesis and Characterization of Hybrid MAuNPs

Previously, we systematically studied the process of obtaining hybrid magnetic Fe_3_O_4_@Au nanoparticles of the “core–satellite” type (MAuNPs), where iron oxide nanoparticles served as the core and gold nanoparticles as satellites [31]. The Fe_3_O_4_@Au core–satellite morphology is characterized by single or multiple magnetite (Fe_3_O_4_) cores surrounded by a distribution of distinct gold (Au) nanoparticles. The gold coating does not cover the entire Fe_3_O_4_ component surface, forming a satellite-type layer all around. The main advantage of such morphology is that it provides the possibility of the further functionalization of both the gold surface and the uncovered Fe_3_O_4_ surface. Furthermore, the core–satellite architecture demonstrates superior saturation magnetization compared to its core–shell counterparts. In contrast to the core–satellite configuration, the core–shell architecture displays reduced saturation magnetization—attributed to the more pronounced diamagnetic contribution of the gold phase—coupled with enhanced colloidal stability and biocompatibility in aqueous environments [32]. We selected nanoparticles with a core–satellite structure because their magnetic properties are critically important for the development of the proposed assay. As the basis for our methodology of forming a satellite-type gold layer on the surface of Fe_3_O_4_ NPs, we selected the synthetic scheme of HAuCl_4_ reduction with sodium citrate [33]. The formation of MAuNPs was carried out in successive stages: (1) synthesis of stabilized Fe_3_O_4_ NPs, (2) controlled growth of gold nanoparticle seeds, (3) addition of stabilized Fe_3_O_4_ NPs to the gold nanoparticle seed solution, (4) formation of the final MAuNPs. Fe_3_O_4_ NPs were obtained by co-precipitation of iron (II) and iron (III) chlorides in a molar ratio of 1:2 in the presence of 0.7 M NH_4_OH [34]. A 2 M HClO_4_ solution was used as a stabilizer for Fe_3_O_4_ NPs, enhancing the electrostatic repulsion between MNPs due to the adsorption of perchlorate ions on the NP surface [35]. The controlled growth of the gold nanoparticle seeds was carried out by maintaining HAuCl_4_ for 5 min in a boiling 0.08 M sodium citrate solution, followed by the addition of freshly prepared stabilized Fe_3_O_4_ NPs. At the final stage, the growth of Au NPs on the surface of Fe_3_O_4_ NPs occurred due to the ongoing exhaustive reduction of HAuCl_4_ by sodium citrate.

The dynamic light scattering (DLS) method showed that the initial Fe_3_O_4_ NPs have a positive surface ζ-potential of 32.6 ± 3.9 mV. The ζ-potential value of the hybrid MAuNPs decreases significantly compared to Fe_3_O_4_ NPs, amounting to 4 ± 5 mV, which is explained by the solvation of negatively charged citrate anions around Au clusters on the Fe_3_O_4_ NP surface. The elemental composition of the synthesized MAuNPs was analyzed by energy-dispersive X-ray spectroscopy (EDS) (Figure 1A). The joint presence of Fe signals (6.4 keV, K-line) and Au signals (2.1 keV, L-line) was observed, which is consistent with the data in the literature for Fe_3_O_4_@Au NPs [36,37]. The Fe content was 32.44 ± 2.81 wt. %, and the Au content was 33.16 ± 3.32 wt.%. These data, together with the calculation of the yield of the final MAuNPs based on gold content (Supplementary Materials), indicate the completeness of AuCl_4_^−^ reduction to Au^0^ within the MAuNPs. The elemental mapping data of the Fe_3_O_4_@Au NP samples demonstrate a uniform distribution of Au and Fe signals over the entire sample area, which indirectly confirms the “core–satellite” structure of the obtained MAuNPs (Figure 1B).

The Fe_3_O_4_@Au NP samples obtained in this study represented (according to TEM data) clusters of iron oxide nanoparticles with diameters of approximately 8–10 nm; on the surface of these, gold inclusions with diameters ranging from 10 to 50 nm were present (Figure 1C). It is particularly noteworthy that the preparation contained neither separately located Au nanoparticles nor clusters of iron oxide nanoparticles devoid of Au nanoparticles.

The MAuNPs were characterized by spectral methods. The IR spectrum of the MAuNPs exhibits two incompletely resolved peaks at 590 and 631 cm^−1^ (Figure 1D). According to data in the literature [38,39], the IR spectrum of magnetite demonstrates two intense infrared absorption bands at 570 and 390 cm^−1^ [40], while maghemite exhibits bands at 630, 590, and 430 cm^−1^ [41]. Consequently, the MAuNPs consist of a mixture of magnetite and maghemite, in analogy with the work of [39]. Based on the lower standard electrode potential of Fe^3+^/Fe^2+^ compared to Au^3+^/Au (E^0^(Fe^3+^/Fe^2+^) = +0.77 V [42], E^0^(Au^3+^/Au) = +1.52 V [43]), it can be stated that the oxidation of Fe^2+^ to Fe^3+^ occurs not only due to contact with atmospheric oxygen but also under the action of AuCl_4_^−^ ions, with the accumulation of the oxidized iron form, Fe_2_O_3_. In the future, this may affect the shelf life of these MAuNPs.

The optical absorption spectrum of the MAuNPs exhibited a peak with a maximum at 567 nm, characteristic of gold nanoparticles [37,44,45] (Figure 1E).

To determine the qualitative phase composition, we analyzed the MAuNPs by X-ray diffraction (XRD). The diffraction patterns (Figure 1F) revealed reflections corresponding to two crystalline phases: metallic gold with a face-centered cubic lattice (JCPDS No. 04-0784) and magnetite Fe_3_O_4_ with a spinel structure (JCPDS No. 19-0629).

The magnetic properties of the MAuNPs were confirmed; we observed the formation of a visible precipitate upon application of a magnetic field in less than 1 min (Figure 1E, inset). The relaxivity values of the MAuNPs, r_1_ and r_2_, were 0.1729 and 2.0639 mmol^−1^·s^−1^, respectively, and the r_2_/r_1_ ratio was 11.9, indicating the T_2_-weighted behavior of the MAuNPs as a potential MRI contrast agent, which is consistent with the data in the literature for Fe_3_O_4_ NPs [46].

Based on the totality of all analytical data, it can be concluded that we have obtained well-characterized hybrid MAuNPs that combine the chemical properties of gold and the magnetic properties of iron oxide, representing a highly promising nanomaterial for a wide range of diagnostic and therapeutic applications.

### 2.2. DNA Target and Probe System

In this work, we used DNA targets—extended (ML, WL), with a length of 198 bp, and short (M, W), with a length of 34 nt—based on a fragment of the *gyrA* gene of *Mycobacterium tuberculosis* (MTB). The extended DNA targets, ML and WT, were obtained according to the scheme in Figure S1 by template-directed ligation using T4 DNA ligase. The coding strand of this fragment in mutant MTB strains contained a single-nucleotide substitution A/C, which confers MTB resistance to fluoroquinolones [47].

The short targets (M and W) and probes (P and Pm) as a model system were previously tested by us in [48] (Figure 2). The length and nucleotide composition of the probes (P and Pm) were selected so that they were capable of forming tandem duplexes (M/P+Pm and W/P+Pm) and incomplete duplexes (M/P, M/Pm, W/P, and W/Pm). The single-nucleotide substitution led to the formation of a mismatch in the duplexes (W/P+Pm) and (W/Pm). The probes carried a functional group at one of their ends for efficient noncovalent adsorption onto the surface of AuNPs or MAuNPs. For this purpose, we used (1) a Cy5 fluorophore residue and a lipotrityl group (Figure S2) or (2) an amino group for conjugation with BSA. The choice of functional groups (1) or (2) in the probe composition was determined by the conditions of their hybridization with the extended (ML and WL) or short (M and W) targets.

The thermodynamic stability of the duplexes formed upon the interaction of each possible target–probe pair, at various DNA concentrations and ionic strengths of the solutions, was previously investigated by us in [48]. Technically, the melting temperature (Tm) values of all analyzed duplexes can be estimated at concentrations no lower than 0.3 μM. Given the need to detect mismatches in the nanomolar range of target concentrations, the Tm values of the studied duplexes at a concentration of 3 nM in 3 mM PBS were determined by computational methods using OligoCalc software v.3.27 in the salt-adjusted mode [49]. The Tm values for the M/Pm and W/Pm duplexes were 57.1 and 54.6 °C, respectively, which allowed us to establish conditions for reliable discrimination of the short DNA targets, M and W, using AuNPs [48]. Based on these data, we developed a methodology for mismatch detection, employing not only AuNPs but also hybrid MAuNPs, in conjunction with the extended targets, ML and WL.

Two schemes were proposed for the detection of SNPs within DNA targets. The first scheme included the following stages (Figure 3A):

1. Addition of a pair of probes to the target under conditions favoring the preferential formation of only the perfect tandem duplex; in the case of extended double-stranded (ds) targets, this was preceded by a denaturation step at 95 °C to release the coding strand and make it accessible for hybridization with the probes.
2. Addition of AuNPs and a buffer solution to create conditions for the formation of sandwich nanostructures containing two AuNPs with tandem duplexes of the target and two probes adsorbed onto them, with different efficiencies for the two targets, ML and WL.

**Figure 3:**
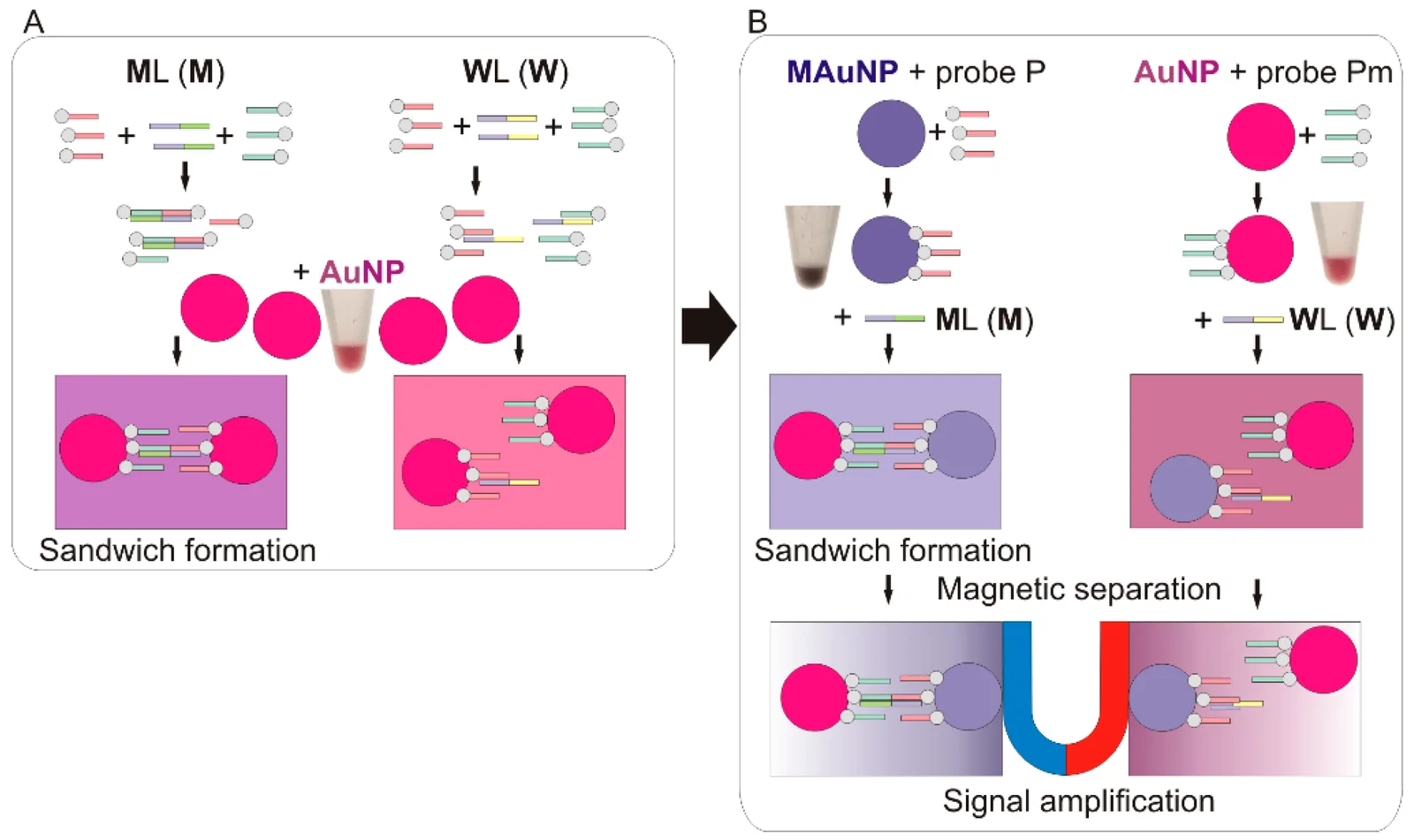
SNP detection schemes. (**A**) Formation of duplexes by the target M (mutant) or W (wild-type) with both probes, P and Pm, at the first stage, followed by the addition of gold nanoparticles (AuNPs). The formation of tandem duplexes by the probes with the M target leads to the proximity of two AuNPs, resulting in the formation of a sandwich structure with different efficiencies for the two different targets. (**B**) Separate preparation of AuNP complexes with probe Pm and magnetic Fe_3_O_4_@Au NP complexes with probe P at the first stage, addition of one of the two targets, M or W, with the formation of a sandwich structure upon adsorption of the resulting tandem duplexes onto the NPs, followed by the application of a magnetic field to enhance the difference in the analytical signal. Different colors correspond to different DNA sequences.

This scheme was optimal for using AuNPs as a colorimetric component due to the possibility for control. The second detection scheme, adapted for the combined use of AuNPs with MAuNPs, consisted of the following steps:

1. Preparation of separate conjugates of AuNPs or MAuNPs with each probe.
2. Addition of the AuNP or MAuNPs conjugates with each probe to one of the two targets at a defined ionic strength and temperature, which promoted the discrimination of the incorrect target; in the case of extended ds-targets, this was preceded by a denaturation step at 95 °C to release the coding strand and make it accessible for hybridization with the probes.

The advantage of the second detection scheme (when using MAuNPs) was the possible enhancement of the analytical signal due to the response of the samples to the application of an external magnetic field (Figure 3B).

The application of either of these two detection schemes leads to the formation of tandem duplexes between the target and two probes and the formation of incomplete duplexes between the target and one of the probes, with different efficiencies for the M and W targets. The formation of a tandem duplex with its subsequent adsorption onto NPs inevitably leads to the proximity of these two NPs. This construct is referred to as a sandwich structure [50].

The different efficiencies of tandem duplex formation for the wild-type and mutant targets due to the presence of a mismatch lead to a difference in the proportion of sandwich structures formed in the samples. When only AuNPs are used for detection, sandwich formation leads to a shift of the plasmon resonance peak to the red region, resulting in visual and spectral colorimetric differences between the samples of the two target types. When AuNPs and MAuNPs are used, sandwich formation also leads to a red shift in the optical absorption spectrum, taking into account that all samples containing both types of nanoparticles simultaneously have a bluer color compared to samples containing only AuNPs.

In this work, we proceeded step by step. First, we studied the applicability of the first scheme using only AuNPs (Figure 3A), which we had previously successfully tested on short, single-stranded DNA targets, M and W (34 nt) [48], to the extended, double-stranded DNA targets, ML and WL (198 bp), which carry the same probe-hybridization sites as the short DNA targets. Next, we tested the possibility of forming a sandwich structure from AuNPs and MAuNPs with probes adsorbed onto them, using the short DNA targets, M and W, and the possibility of enhancing the degree of target discrimination through the precipitation of the formed sandwich structures under the action of an external magnetic field (Figure 3B). Finally, we adapted the assembly of the sandwich structure from AuNPs and MAuNPs to the samples of the extended DNA targets, ML and WL.

### 2.3. SNP Detection in Extended Targets Using AuNPs

At the first stage, we tested the possibility of SNP detection in extended DNA targets (198 bp) using only AuNPs and adapting our previous experience. We used the well-known property of the strong noncovalent adsorption of native [51,52,53,54,55] and fluorescently labeled or hydrophobically modified [56] oligonucleotides onto the surface of AuNPs and other inorganic NPs [57,58]. Probe P carried a Cy5 residue at the 3′-end, since we previously showed that this fluorophore has a high affinity for AuNPs and is capable, under certain conditions (including molar ratios of NPs and Cy5-labeled probes, incubation time and temperature), of ensuring 100% adsorption of such probes onto AuNPs [56]. The second probe, Pm, carried a lipotrityl hydrophobic group at the 5′-end, which also exhibits high affinity for AuNPs, according to [48].

It should be noted that the development of colorimetric detection does not require the maximum loading of NPs with probes, since excessive probe surface density (the number of probe molecules per one AuNP) hinders the proximity of NPs to each other and the formation of the colorimetric response required for detection [59]. Therefore, we worked in the loading range of 5 to 20 probe molecules per AuNP.

It is known that there is competition between the processes of (1) the formation of the target/probe duplex and (2) the adsorption of the duplex itself and its nucleotide components onto AuNPs separately [60,61]. Since this can uncontrollably affect the colloidal stability of AuNPs and the analytical signal associated with its fluctuations [62], we applied the single-nucleotide mismatch-detection schemes (Figure 3), which eliminate this competition, by analogy with [63].

To create the required ionic strength and enable duplex formation, we used SSC buffer solution (to final concentrations of 56 mM NaCl, 5 mM Na_3_C_6_H_5_O_7_, pH 7.4) [64]. Discrimination of the formation of tandem duplexes, perfect and containing a single-nucleotide mismatch, was carried out for 40 min at 54 °C, which was selected according to [65].

The protocol developed based on the scheme (Figure 3A) was tested in a series of three independent experiments with four replicates each. To evaluate the detection results, visual analysis (photographs of samples) and optical absorption spectroscopy were used, with calculation of the optical density ratio at 520 nm (absorption maximum of AuNPs) and at 620 nm (corresponding to the absorption of aggregated AuNPs) (Figure 4). Typical optical absorption spectra are presented in Figure S3. As a result, we revealed a reliable colorimetric difference between the samples with the wild-type and mutant targets (*p*-value 0.0007) at a DNA target concentration of 3 nM, unlike the published method for SNP detection in exon 5 of the human cytochrome P450 2C19 monooxygenase gene at micromolar concentrations requiring preliminary amplification [66].

Through visual and spectral analysis, the formation of sandwich structures from a pair of AuNPs with adsorbed tandem duplexes of the probes with the mutant target was established, which were spectrally distinct from individual AuNPs with adsorbed incomplete duplexes of the probes with the wild-type target. Thus, the applicability of AuNPs with noncovalently adsorbed probes for mismatch detection in extended DNA targets was demonstrated. This result was subsequently used to enhance the analytical signal by employing the magnetic MAuNPs obtained in this study.

### 2.4. SNP Detection in Short Targets Using AuNPs and MAuNPs

Next, SNP detection was carried out using two types of NPs (AuNPs and MAuNPs). The assay protocol was optimized on short 34-nt single-stranded DNA targets and probes containing a Cy5 residue or a lipotrityl group. As we previously showed in [48], colorimetric sensors based on AuNPs are characterized by high sensitivity due to their high extinction coefficient; however, they are susceptible to a decrease in colloidal stability upon fluctuations in the ionic strength of the solution. The use of MAuNPs together with AuNPs makes it possible to avoid high ionic strength values for the solution and thereby preserves the colloidal stability of the system [67]. This also makes it possible to separate the formed sandwich constructs from the reaction mixture by concentrating them on a magnet, leading to a more pronounced colorimetric difference between the target samples and the mismatch-containing samples, by analogy with antibody-based sensors using AuNPs and Fe_3_O_4_@Au NPs [27,68,69]. Methods employing hybrid MAuNPs are primarily aimed at detecting target antigens using antibodies—such as serum amyloid A (SAA), C-reactive protein [69], interleukin-6 [70], and the CFP-10 antigen associated with MTB [26,27]. The proposed assay uses DNA probes instead of antibodies as a more stable and less expensive alternative.

To apply MAuNPs in the SNP detection system, it was necessary to evaluate their adsorption capacity with respect to the DNA probes used. Upon incubation of Fe_3_O_4_@AuNPs with the DNA probe P-Cy5 in the range of 6.25–1500 pmol of probe per 1 mg of MAuNPs at 25 °C for 1 h, 100% adsorption efficiency was established. The maximum value was 1500 ± 1 pmol of probe per 1 mg of MAuNPs, which exceeds the corresponding value for AuNPs—834 ± 30 pmol of probe per 1 mg of AuNPs (56% adsorption efficiency). It is known that both the gold surface and the Fe_3_O_4_ surface are capable of efficient noncovalent adsorption of DNA, similar to other inorganic NPs [71,72]. This is explained by the larger specific surface area of hybrid MAuNPs compared to single-component AuNPs relative to the NP mass. For the development of the detection system, a loading level of MAuNPs in the range of 5–20 molecules per nanoparticle (or 111–444 pmol of probe/1 mg of MAuNPs) is acceptable, as in the case of AuNPs.

To control the colloidal stability of any NPs, protection of their surface from undesirable/non-target aggregation, including salt-induced aggregation, is required. However, excessive stabilization of NPs leads to overly strong electrostatic repulsion forces between NPs, preventing them from approaching each other, since they are shielded by the solvation shell of these compounds, and, as a consequence, they cannot form a sandwich structure [59]. NP surfaces are stabilized with high-molecular-weight compounds (surfactants, proteins, carbohydrates, Tween-20, BSA, dextran) and low-molecular-weight compounds [73,74,75]. It should be taken into account that DNA adsorbs poorly onto AuNPs coated with high-molecular-weight compounds (e.g., proteins) and has low hybridization ability [76]. Therefore, we used fluorescein, which has the ability to adsorb rapidly and efficiently onto the AuNP surface, according to [56]. We found that the dosed, precisely adjusted addition of fluorescein to AuNPs (at a molar ratio of 100:1 to AuNPs) stabilizes the AuNP surface, making it sufficiently protected from non-target salt-induced aggregation while, at the same time, leaving it capable of approaching other NPs and forming a sandwich structure with a corresponding colorimetric response. Detection was carried out according to the scheme in Figure 3B. To create the required ionic strength, we used SSC buffer solution (to final concentrations of 56 mM NaCl, 5 mM Na_3_C_6_H_5_O_7_, pH 7.4). For detection, the optical absorbances at 520 nm and 650 nm were recorded, and their ratio was calculated (Figure 5).

We observed different shades of the samples: redder for the M target and bluer for the W target at a target concentration of 7 nM. Sandwich formation, as discussed above, is made possible by complementary interactions of the probes with the template and by the proximity of NPs to each other due to the adsorption of tandem duplexes onto them. The proximity of the two types of NPs to each other leads to irreversible aggregation, as shown for the sample containing no target (Figure S4). The formation of sandwich structures occurs with different efficiencies for targets differing by a single SNP. The application of an external magnetic field to the samples leads to the removal of a portion of NPs from the solution, with precipitation of a certain fraction of both sandwich structures and individual MAuNPs onto the magnet. Sandwich structures, due to the presence of AuNPs, have more intense optical absorption compared to MAuNPs; therefore, the removal of even a fraction of them leads to a detectable decrease in the color intensity of the solution. As a consequence, an enhancement of the colorimetric difference between the M and W target samples is observed (Figure 5). In this case, the visual analysis becomes more evident, and the difference between the spectral D_520_/D_650_ ratios also increases.

Thus, the use of MAuNPs together with AuNPs for SNP detection in short DNA targets M and W makes it possible to apply a magnetic field to the samples, which increases the spectral difference between them and makes their differentiation more reliable: the *p*-value decreases from 2.748 × 10^−6^ to 3.755 × 10^−12^.

### 2.5. SNP Detection in Extended Targets Using AuNPs and MAuNPs

The use of MAuNPs together with AuNPs for SNP detection in the extended targets, ML and WL, required the optimization of the previously tested detection protocols. We found that the simultaneous addition of both types of NPs with adsorbed probes carrying hydrophobic groups for efficient adsorption onto NPs (Cy5 or a lipotrityl group) to the extended targets led to NP aggregation, with each being faster than the formation of tandem duplexes between the probes and the target. To preserve the colloidal stability of NPs, prevent their non-target aggregation during detection, and improve the accuracy of the colorimetric response in the case of extended DNA templates, an alternative approach to probe adsorption onto the surface of AuNPs and MAuNPs was chosen. It consisted of conjugating each probe with a BSA molecule [77] and adsorbing the resulting conjugates onto the surface of AuNPs and Fe_3_O_4_@AuNPs. This variant of probe modification solves two problems: it ensures efficient adsorption of probes onto AuNPs and MAuNPs and protects NPs from non-target aggregation. The degree of conversion upon the conjugation of BSA with the P-NH_2_ probe was approximately 67% (according to the analysis of the electrophoretic mobility of the conjugates and initial BSA by SDS-PAGE). Under the analysis conditions we selected (54 °C), the secondary structure of BSA within the BSA/probe conjugates did not differ from the structure of the free protein (Figure S5). The adsorption of the conjugates onto NPs (AuNPs or MAuNPs) was carried out for 1 h at 25 °C at a molar ratio of conjugates to AuNPs of 100:1. This ensured a conjugate-loading capacity onto the AuNP surface of approximately 15 molecules per AuNP, or 333 pmol per 1 mg of MAuNPs, which correlates with the data in the literature on BSA adsorption onto AuNPs [51]. The previously proposed SNP-detection scheme for the extended targets ML and WL (Figure 3B) was adjusted as follows: (1) target samples were subjected to annealing at 95 °C, (2) then AuNPs with adsorbed probe Pm were added first, (3) followed by MAuNPs with adsorbed probe P, and the mixture was incubated under the required conditions to promote the formation of the tandem duplex between the template and the probes. For SNP detection, the optical absorbance at 570 nm and 650 nm was analyzed, and their ratio was calculated (Figure 6).

We observed a colorimetric difference between the two targets at a concentration of 8 nM. The application of an external magnetic field led to a more pronounced collection of sandwich constructs onto the magnet in the sample with the ML template compared to WL. The calculation of the spectral D_570_/D_650_ ratios for the studied samples confirmed this observation (Figure 6). The spectral D_570_/D_650_ ratio for the samples with the ML and WL targets differs from the cases where the sandwich structure was not formed (with a noncomplementary target, see the control template in Figure 6 and Figure S4) or where its proportion was extremely small. In this case, under the action of an external magnetic field, only MAuNPs will precipitate, resulting in a red-colored supernatant. These observations confirm the depletion of AuNPs from the reaction mixture precisely due to the formation of sandwich structures in the case of the ML target. It should be noted that, both in the absence of any target and in the presence of a non-complementary target, no relevant analytical signal is observed, which confirms the specificity of our method (Figure S4).

In the sample containing the non-complementary template, a redder shade is observed than in the sample with the ML target, and the spectral pattern is similar to that of the WL sample, corresponding to the absence of sandwich structure formation. In the sample containing no template, AuNPs stick together with a loss of their colloidal stability, and a pronounced blue shade is observed.

The composition of the supernatants of the samples with the two targets after application of the external magnet was investigated by TEM (Figure 7). In the samples of both targets, clusters of AuNPs were observed; however, their size and number differ significantly for the ML and WL targets. In the samples containing the WL target, these clusters occur more frequently and contain a greater number of AuNPs than in the samples with the ML target. It can be concluded that a greater depletion of AuNPs from the ML samples occurs under the action of the magnetic field compared to the WL samples. This phenomenon can only occur as a result of the more efficient formation of sandwich structures between the tandem duplexes, AuNPs, and MAuNPs.

Thus, a protocol was developed for the assembly of sandwich constructs based on AuNPs and hybrid MAuNPs with DNA probes for the detection of a single-nucleotide mutation in extended (198 bp) DNA templates at the nanomolar concentration level. The conditions for the discrimination of wild-type and mutant DNA targets using this sandwich system were established. The colloidal stability of the nanoparticles (AuNPs and Fe_3_O_4_@AuNPs) constituting the sandwich system was monitored at all stages of the detection process. The detection protocol takes no longer than 15 min. It was shown that the GNP aggregation process during the adsorption of a preformed duplex takes 3–4 min [78]. Thus, the possibility of reducing the detection protocol to 15 min is consistent with the data in the literature.

A number of colorimetric methods for SNP detection have been published [78,79,80]. These studies achieve very low (0.05 nM) SNP-detection levels, but only in short model ssDNA targets. These studies demonstrated the effect of a significant, by 3 orders of magnitude, reduction in the level of mutation detection when using GNPs of increased diameter (up to 63 nm). For example, colorimetric SNP detection in the BRCA1 gene associated with breast and ovarian cancer was conducted using 13, 46, and 63 nm AuNPs. A 5-fold increase in AuNP size improved the detection limit, reaching 0.05 nM in short ssDNA targets (19 nt) within 10 min [79]. A 1–2 order-of-magnitude reduction in the SNP-detection limit in short synthetic ssDNA targets (15 nt)—down to 1 nM—was achieved by using AuNPs with diameters of 30, 40, and 50 nm instead of 15 nm [80]. When switching to double-stranded extended DNA fragments, these methods will inevitably need to be adapted and a target denaturation step should be added. This will lengthen the detection protocol and will likely complicate the preparation of AuNP associates with probes. Our approach also starts with short ssDNA targets (34 nt), ultimately achieving SNP detection in extended dsDNA targets (198 bp), which is its undoubted competitive advantage. The colorimetric paper-based method [21] allows for the detection of MTB DNA in purified samples within 60 min, which is four times the duration of the proposed approach. The preparation of a genomic DNA sample from a clinical specimen, such as sputum, takes approximately 1–1.5 h. Thus, when moving to clinical bacterial samples, the complete analysis protocol will take 1.25–1.5 h. The use of larger diameter AuNPs can potentially reduce the detection limit in the presented approach, which provides prospects for its further development.

## 3. Materials and Methods

### 3.1. Reagents and Chemicals

Tetrachloroauric acid (HAuCl_4_∙4H_2_O) was purchased from Aurat (Moscow, Russia); sodium citrate dehydrate (Na_3_C_6_H_5_O_7_), 1-Ethyl-3-(3-dimethylaminopropyl)carbodiimide, and bovine serum albumin were purchased from Sigma–Aldrich (Darmstadt, Germany); ethidium bromide was purchased from Acros (Princeton, NJ, USA); Tris was purchased from Suzhou Yacoo Science (Suzhou, China); H_3_BO_3_ was bought from Soyuzkhimprom (Novosibirsk, Russia); acetonitrile was purchased from RCI Labscan (Bangkok, Thailand); Cy5 NHS ester and fluorescein isothiocyanate were from Lumiprobe (Moscow, Russia); NaCl was from Honeywell (Seelze, Germany); DMSO was from Serva (Heidelberg, Germany); C_2_H_5_OH was from Kemerovo Pharmaceutical Factory (Kemerovo, Russia); agarose was from Lonza (Rockland, NY, USA); glycine was from Amresco Chemicals (Zottegem, Belgium); phenol, chloroform, hydrochloric acid, perchloric acid, and a 30% aqueous ammonia solution were acquired from Reakhim (Moscow, Russia); Tween 20 was from Solarbio (Beijing, China); triethylamine (99.5%), iron chloride (II), and iron chloride (III) were from Panreac (Castellar del Vallès, Spain); T4 DNA ligase was purchased from EvroGen (Moscow, Russia); sodium hydrogen phosphate dodecahydrate was from Alfachem (Sankt Peterburg, Russia); sodium dihydrogen phosphate dihydrate was from Reatex (Moscow, Russia); polyethylene glycol 2 kDa was from CDH (New Delhi, India). Water was purified by means of a Simplicity 185 system (Millipore, Burlington, MA, USA) and had a resistivity of 18.2 MW cm at 25 °C. Control DNA was kindly provided by Dr. Elena Dmitrienko (ICBFM).

### 3.2. Synthesis of DNAs

Unmodified oligonucleotides and their derivatives containing an amino group or fluoresceine at the 5′ or 3′ end (Table 1) were synthesized on an ASM-800 (Biosset, Novosibirsk, Russia) following the solid-phase phosphoramidite protocol using DNA phosphoramidites from ChemGenes (Wilmington, MA, USA).

Lipo in the oligonucleotides has the structure shown in Figure S2. DNA Lipo-Pm was synthesized and purified as described in [48,81].

DNAs M, W, F1, F2, FW3, FM3, F4, R1, R2, RM3, RW3, R4, and R5 were purified by electrophoresis in a 10% polyacrylamide gel containing 8 M urea (10% PAAG). Electrophoresis was carried out in TBE (89 mM Tris, 89 mM H_3_BO_3_, 2 mM EDTA, pH 8.5) for 90 min at 20 V/cm.

DNA P-NH_2_ was fluorescently labeled at the 3′ end using a method described in [52] and then was purified by electrophoresis in 15% PAAG containing 8 M urea.

DNAs NH_2_-L_18_-Pm and P-L_18_-NH_2_ were purified by reversed-phase high-performance liquid chromatography (RP HPLC) on an Agilent 1200 Series using a Zorbax 5 μm Eclipse-XDB-C18 80 Å column (150 × 4.6 mm^2^) from Agilent Technologies (Santa Clara, CA, USA), via a linear gradient of acetonitrile in 0.02 M TEA-Ac (0–50%) during 30 min.

The optical density of DNA solutions was determined using a spectrophotometer NanoDrop One C (ThermoFisher Scientific, Emeryville, CA, USA). Concentrations of all DNAs were determined using molar coefficients of absorption of the dinucleotides at a wavelength of 260 nm [82].

### 3.3. Synthesis of AuNPs

AuNPs were synthesized according to the classic citrate reduction method [83]. The size of AuNPs was determined using a JEM-1400 transmission electron microscope (Jeol, Tokyo, Japan) equipped with a Veleta digital camera (EM SIS, Münster, Germany).

Hydrodynamic diameter and ζ-potential of AuNPs as well as their dispersity were evaluated by dynamic light scattering technique using a Zetasizer Nano ZS Plus instrument (Malvern Instruments, Malvern, UK). The concentration of AuNPs was determined from absorbance at 520 nm using an extinction coefficient of (8.78 ± 0.06) × 10^8^ M^−1^ cm^−1^ [44].

### 3.4. Synthesis of MNPs Fe_3_O_4_

Weighed samples of FeCl_3_∙6H_2_O (1.082 g) and FeCl_2_∙4H_2_O (0.398 g) were dissolved in 5 mL of 2 M HCl. The resulting iron salt solution was added to 50 mL of 0.7 M NH_4_OH solution and stirred for 30 min at 25 °C. The Fe_3_O_4_ NPs were washed twice with 2 M perchloric acid using magnetic separation. The resulting NPs were dispersed in 45 mL of 2 M perchloric acid.

### 3.5. Synthesis of MAuNPs Fe_3_O_4_@Au

A flask containing 26.25 mL of H_2_O mQ was added with 3.75 mL of a 4.85∙10^−3^ M HAuCl_4_∙4H_2_O solution and brought to a boil using a magnetic stirrer (DLab, Beijing, China). First, 1.25 mL of a 0.08 M sodium citrate solution was added to the boiling tetrachloroaurate solution. After 5 min, 1.25 mL of freshly prepared Fe_3_O_4_ magnetic nanoparticles was added. The solution was boiled for 15 min and then allowed to cool to room temperature.

### 3.6. Obtaining ML and WL DNA Targets

Solutions of 330 μL volume containing 0.6 μM of each of the oligonucleotides R1, R2, RM3/RW3, R4, R5, F1, F2, FM3/FW/3, F4 (equivalent to 200 pmol of the 5′-terminal phosphate group of each of the ligated oligonucleotides) were heated to 95 °C for 5 min and cooled to 25 °C. Then, 60 μL of 10× reaction buffer solution for overnight ligation from the manufacturer was added, mixed, and 60 μL of T4 DNA ligase (Eurogen, Moscow, Russia) 50 U/μL (i.e., 3000 U) was added. The reaction was carried out at 25 °C and stirring at 800 rpm for 5 days. Proteins were extracted from the reaction mixture with an equal volume of a phenol/chloroform mixture (1:1). The procedure was repeated using an equal volume of chloroform. The ligation products were precipitated as the sodium salt in ethanol, followed by desalting by gel filtration using Micro BioSpin P-30 spin columns (Bio-Rad Laboratories, Hercules, CA, USA). The homogeneity and length of the ligation products were assessed by gel electrophoresis in a 1.5% agarose gel in a 0.5× TBE buffer solution at 95 V for 1 h with visualization of the nucleotide material with ethidium bromide at a concentration of 0.001 mg/mL using VersaDocTM MP 4000 Molecular Imager^®^ System and Quantity One software, version 4.6.9 (Bio-Rad Laboratories, Hercules, CA, USA).

### 3.7. Preparation of BSA and DNA Probe Conjugates

To 1 μL of 10 mg/mL BSA, 1.5 μL of 100 mM PB pH 6.0 and 10 μL of an aqueous solution of 0.1 M EDC were added and mixed. After 1 min, 4 μL of a 100 μM solution of NH_2_-L_18_-Pm or P-L_18_-NH_2_ was added. The solution was then incubated for 2.5 h at 25 °C with stirring at 350 rpm. The conjugates were purified from reaction mixture components by gel filtration using Bio-Rad P-30 spin columns (Bio-Rad Laboratories, Hercules, CA, USA).

### 3.8. Preparation of AuNP/BSA-Pm and MAuNP/BSA-P Associates

To 261 μL of 4.27 nM AuNP (1.1 pmol) or 450 μL of MAuNP 0.1 mg/mL (0.045 mg), 10 μL of 10% PEG-2000 and 2 μL of 10% Tween 20 were added, and the mixture was incubated at 25 °C with stirring at 800 rpm for 20 min. Then, 12.5 μL of the BSA–DNA–probe conjugate solution was added and incubated at 25 °C with stirring at 800 rpm for 1 h. Next, AuNPs/BSA-Pm were isolated from the reaction mixture by centrifugation at 4000× *g* for 30 min, and MAuNPs/BSA-P were precipitated using an external magnet (Helicon, Moscow, Russia), the supernatant was removed, and H_2_O mQ was added to a final solution volume of 231 μL.

### 3.9. Preparation of AuNP/Lipo-Pm and MAuNP/P-Cy5 Associates

To 261 µL of 4.27 nM AuNP (1.1 pmol) or 450 µL of 0.1 mg/mL MAuNP (0.045 mg), 2.25 µL of a 2.5 µM solution of LipoPm or P-Cy5 (5:1 by moles) was added, respectively, and the mixture was incubated at 25 °C with stirring at 800 rpm for 16 h. Then, 1.12 µL of 50 µM FITC was added to the reaction mixture containing AuNP and the mixture was incubated at 25 °C with stirring at 800 rpm for 30 min. Next, AuNPs/Lipo-Pm were isolated from the reaction mixture by centrifugation at 4000× *g* for 30 min, MAuNPs/P-Cy5 were precipitated using an external magnet, and the supernatant was removed. H_2_O was added to AuNPs/Lipo-Pm to a final volume of 231 μL, and 12.8 μL of SSC 20× and H_2_O mQ were added to MAuNPs/P-Cy5 to a final volume of 231 μL.

### 3.10. Calculation of DNA Adsorption Efficiency

Samples (final volume 800 μL) containing 0.08 mg MAuNPs and a 0.5–120 pmol of oligonucleotide Pm-Cy5 were incubated at 25 °C for 1 h. MAuNPs were pellets using the external magnet during 5 min, and the supernatants were collected. The fluorescence intensity of supernatants containing Cy5-labeled oligonucleotide was measured using a Clariostar microplate fluorimeter (BMG Labtech, Ortenberg, Germany). Cy5 fluorescence was recorded with excitation at 610 nm and emission at 675 nm. Based on these measurements, the concentration of unbound oligonucleotide was determined. All measurements were performed at least in quadruplicate. DNA adsorption efficiency (pmol of DNA per 1 mg of MAuNPs) was calculated from fluorescence intensity according to Equation (1):

*n* = (12.5·[DNA]_b_/[DNA]_t_)·100%, (1)

where n is the DNA adsorption efficiency, [DNA]_b_ is the concentration of DNA bound to MAuNPs, and [DNA]_t_ is the total DNA concentration.

### 3.11. SNP Detection Protocol in Extended DNA Targets Using AuNPs

To 102.6 μL of a solution containing 8 nM DNA probes and 0.025× SSC buffer solution (4 mM NaCl, 0.4 mM Na_3_C_6_H_5_O_7_ pH 7.4), 55.4 μL of 15 nM DNA target solution was added and the mixture was incubated first at 95 °C for 5 min, then at 54 °C for 5 min. Next, 98 μL of 2.6 nM AuNPs was added to the mixture and incubated at 54 °C for 10 min. Then, 14.9 μL of 10.5× SSC buffer solution (1580 mM NaCl, 158 mM Na_3_C_6_H_5_O_7_ pH 7.4) was added to the mixture four times, incubating at 54 °C for 10 min between additions. Optical absorption spectra were recorded in the range of 400–800 nm using a Clariostar plate fluorometer (BMG Labtech).

### 3.12. SNP Detection Protocol in Short DNA Targets Using AuNPs and MAuNPs

To 55.4 μL of 0.1 μM DNA target solution, preincubated at 54 °C for 5 min, 25.7 μL of the AuNP/Lipo-Pm associate solution was added successively, followed by the addition of 25.7 μL of MAuNP/P-Cy5, and the mixture was incubated at 54 °C for 10 min. Next, 7 μL of 2× SSC buffer solution (300 mM NaCl, 30 mM Na_3_C_6_H_5_O_7_ pH 7.4) was added, and the mixture was incubated at 54 °C for 10 min. Optical absorption spectra were recorded in the 400–800 nm range using a Clariostar plate fluorometer (BMG Labtech). To study the response to an external magnetic field, samples were placed in a magnetic tube rack for 20 min. After that, the supernatants were collected and their optical absorption spectra were recorded as described above.

### 3.13. SNP Detection Protocol in Extended DNA Targets Using AuNPs and MAuNPs

To 55.4 μL of a 15 nM DNA target solution preincubated at 95 °C for 5 min, 25.7 μL of the AuNP/Lipo-Pm associate solution was added sequentially, followed by MAuNP/P-Cy5, and the mixture was incubated at 54 °C for 5 min. Optical absorption spectra were recorded in the 400–800 nm range using a Clariostar plate fluorometer (BMG Labtech). To study the response to an external magnetic field, samples were placed in a magnetic tube rack for 3 min. After that, the supernatants were collected and their optical absorption spectra were recorded as described above.

### 3.14. UV–Vis Spectroscopy

Optical adsorption spectra of the DNA probes with AuNPs and MAuNPs were recorded in 50 μL aliquots using a Clariostar plate fluorimeter (BMG Labtech, Ortenberg, Germany) in the range of 400–800 nm according to the manufacturer’s instructions.

The optical adsorption spectra of the MAuNPs samples were recorded in 200 μL aliquots using a NanoDrop One C (ThermoFisher Scientific, Emeryville, CA, USA) in the range of 400–800 nm according to the manufacturer’s instructions.

### 3.15. Dynamic Light Scattering (DLS)

MAuNPs and Fe_3_O_4_ NPs were analyzed using a Malvern Zetasizer Nano (Malvern Instruments, Worcestershire, UK), according to the manufacturer’s instructions, to determine the ζ-potential and hydrodynamic diameter of the species. Each measurement was performed for thirty runs.

### 3.16. Transmission Electron Microscopy (TEM)

A measure of 2 μL of the particle suspension was applied to a 200-mesh copper grid coated with formvar film, then the grids were air-dried. Samples were examined using a JEM 1400 transmission electron microscope (JEOL, Tokyo, Japan), and images were collected using a Veleta digital camera (EM SIS, Munster, Germany) and the iTEM software, version 5.2 (EM SIS, Münster, Germany).

### 3.17. Determination of Relaxivities r_1_ and r_2_

The T_1_ and T_2_ relaxation times of MAuNPs samples (0.5 mL) were measured across a range of MAuNPs concentrations using an 80 MHz (1.88 T) Spinsolve 80 NMR spectrometer (Magritek, Aachen, Germany) maintained at 25 °C. The relaxivities r_1_ and r_2_ were calculated according to [46].

### 3.18. Circular Dichroism (CD) Spectra

Circular dichroism spectra were recorded using a Jasco J-600 (Jasco, Tokyo, Japan), and recording was carried out from 185 to 330 nm in a quartz cuvette with an optical path length of 1 cm (Jasco, Tokyo, Japan); the volume of the studied sample was 1 mL and the protein concentration was 3 μM.

### 3.19. X-Ray Diffraction Analysis (XRD)

X-ray diffraction (XRD) analysis of the NPs was performed on a Bruker D8 Advance powder diffractometer (Bruker Corporation, Billerica, MA, USA) (CuKα radiation, Lynx-Eye one-dimensional detector with a nickel filter, 0.1° step size, 35.4 s accumulation time per point).

### 3.20. Energy-Dispersive X-Ray Spectroscopy (EDS)

The elemental composition of the samples (iron, gold) and the distribution of chemical elements were determined by energy-dispersive X-ray spectroscopy (EDS) using a Hitachi 3400 N scanning electron microscope (Hitachi Ltd.,Tokyo, Japan).

### 3.21. Fourier-Transform Infrared Spectroscopy (FTIR)

The IR spectra of the samples were recorded on a 640-IR Fourier Transform IR spectrometer (Varian, Palo Alto, CA, USA) in the wavelength range of 4000 to 400 cm^−1^ at room temperature using a KBr pellet.

### 3.22. Statistics

The data obtained by the methods described in Section 3.11, Section 3.12 and Section 3.13 were statistically processed by Student’s *t* test, and differences with a *p* value 1 of <0.05 were considered statistically significant. Microsoft Excel 2016 software version 2304 (Microsoft Corporation, Redmond, WA, USA) was used for this analysis. The data are presented as a boxplot for at least 8 independent measurements.

## 4. Conclusions

Hybrid magnetic Fe_3_O_4_@Au nanoparticles of the “core–satellite” type were synthesized using a method based on [Lyon] with modifications. The elemental and phase compositions of the obtained Fe_3_O_4_@AuNPs (MAuNPS) were systematically characterized by IR and UV–Vis spectroscopy, X-ray diffraction, and energy-dispersive X-ray spectroscopy. The morphology of the MAuNPs was investigated by transmission electron microscopy. The optical properties, magnetic properties (rate of collection on a magnet and relaxivity), and adsorption capacity with respect to the DNA probes of the synthesized Fe_3_O_4_@Au NP samples were studied. It was established that the MAuNPs exhibit surface plasmon resonance with an optical absorption maximum at 567 nm, characteristic of nanoscale gold, and pronounced magnetic properties, collecting on a magnet in less than 1 min. The high adsorption capacity of the MAuNPs with respect to DNA probes was revealed, reaching 1500 pmol of probe per 1 mg of MAuNPs.

A scheme for the assembly of sandwich structures containing AuNPs, MAuNPs, and DNA probes was developed for the detection of a single-nucleotide mutation within an extended DNA target (198 bp), based on the methodology for detecting a single-nucleotide mutation in short DNA targets using only AuNPs, which we previously presented in [48]. The DNA probes were noncovalently adsorbed onto the surface of AuNPs and MAuNPs using two approaches: via hydrophobic residues of the Cy5 fluorophore or a lipotrityl group, or as part of a conjugate with BSA, which serves both as an adsorption anchor and as a stabilizer of the NP surface. The proposed protocol enables the detection of a single-nucleotide mutation by colorimetric detection at the nanomolar level of DNA target concentrations. Visual analysis and optical absorption spectroscopy were used to evaluate the detection results. The detection time was no longer than 40 min. The possibility of concentrating the formed target sandwich constructs using an external magnetic field was demonstrated, which allowed for enhancement of the analytical signal. As a consequence, this led to a more pronounced differentiation of the two targets.

The proposed approach, combining the colorimetric and magnetic potential of AuNPs and MAuNPs, can be successfully adapted for the colorimetric detection of other nucleic acid targets. Potential limitations of the method for adapting to other targets are related to the need for a sufficient difference in the efficiency of tandem duplex formation between the wild-type and mutant targets. This is determined by the mutation itself and its nucleotide environment, the GC composition of the targets and probes, and their length. The enrichment of other target analytes under the action of an external magnetic field may find its niche for enhancing the colorimetric signal and for the sample-preparation step in other analytical methods (ELISA, immunoassays, etc.).

## Figures and Tables

**Figure 1:**
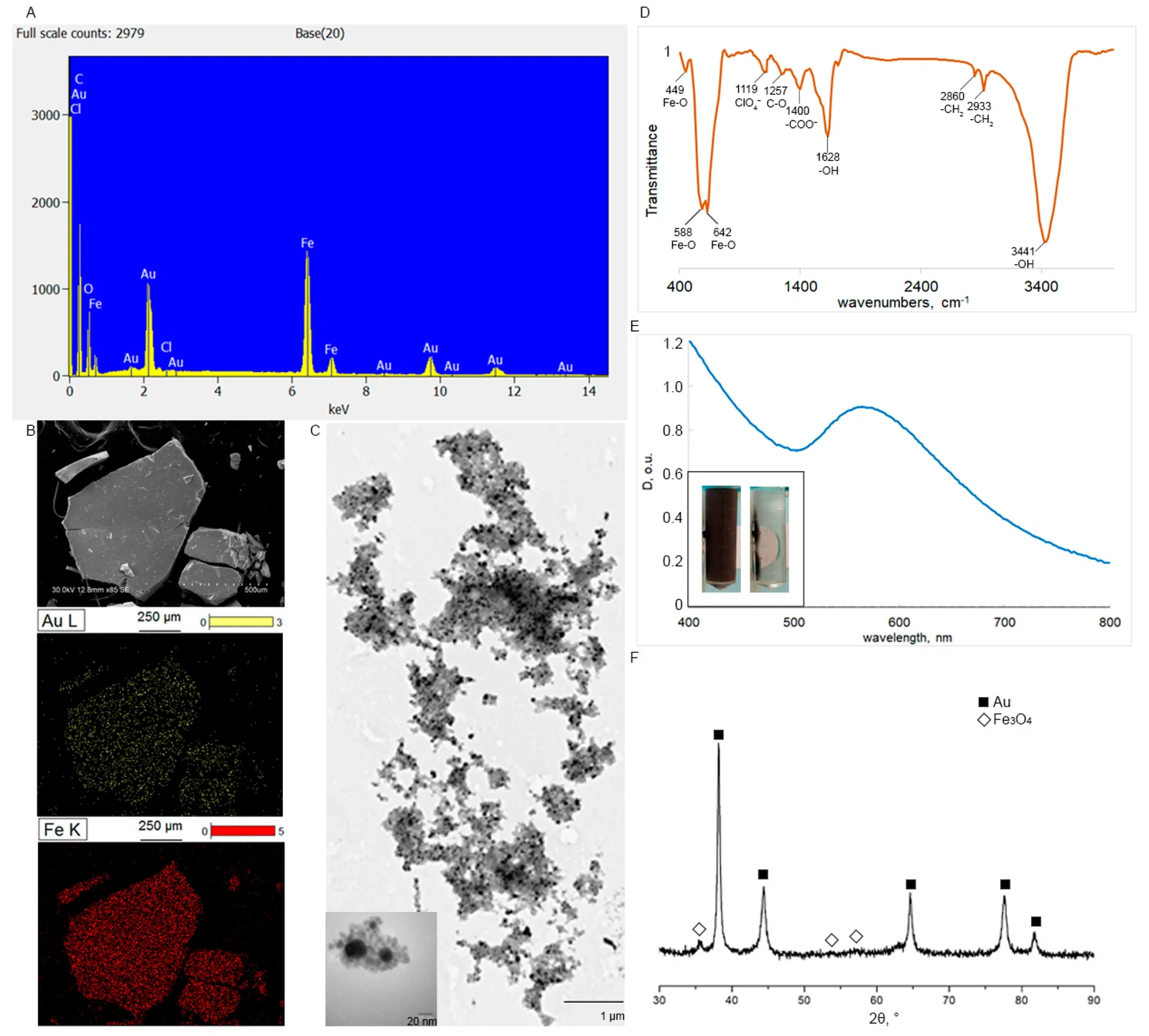
Characterization of MAuNPs by physicochemical methods. Analysis of the Fe_3_O_4_@Au NP sample by EDX method: (**A**) spectrum; (**B**) elemental mapping of nanoparticles. Data were obtained by EDX using a scanning electron microscope. (**C**) TEM images and size distribution of MAuNPs; the length of the scale bar is indicated in each image. (**D**) Infrared spectra of the Fe_3_O_4_@Au NP sample. (**E**) Absorption spectroscopy of synthesized MAuNPs; inset shows a view of the Fe_3_O_4_@Au NP reaction mixtures before (left) and after (right) collection on a magnet for 1 min. (**F**) X-ray diffraction patterns of MAuNPs. The peaks corresponding to Au and Fe_3_O_4_ were identified according to (JCPDS No. 04-0784) and (JCPDS No. 19-0629), respectively.

**Figure 2:**
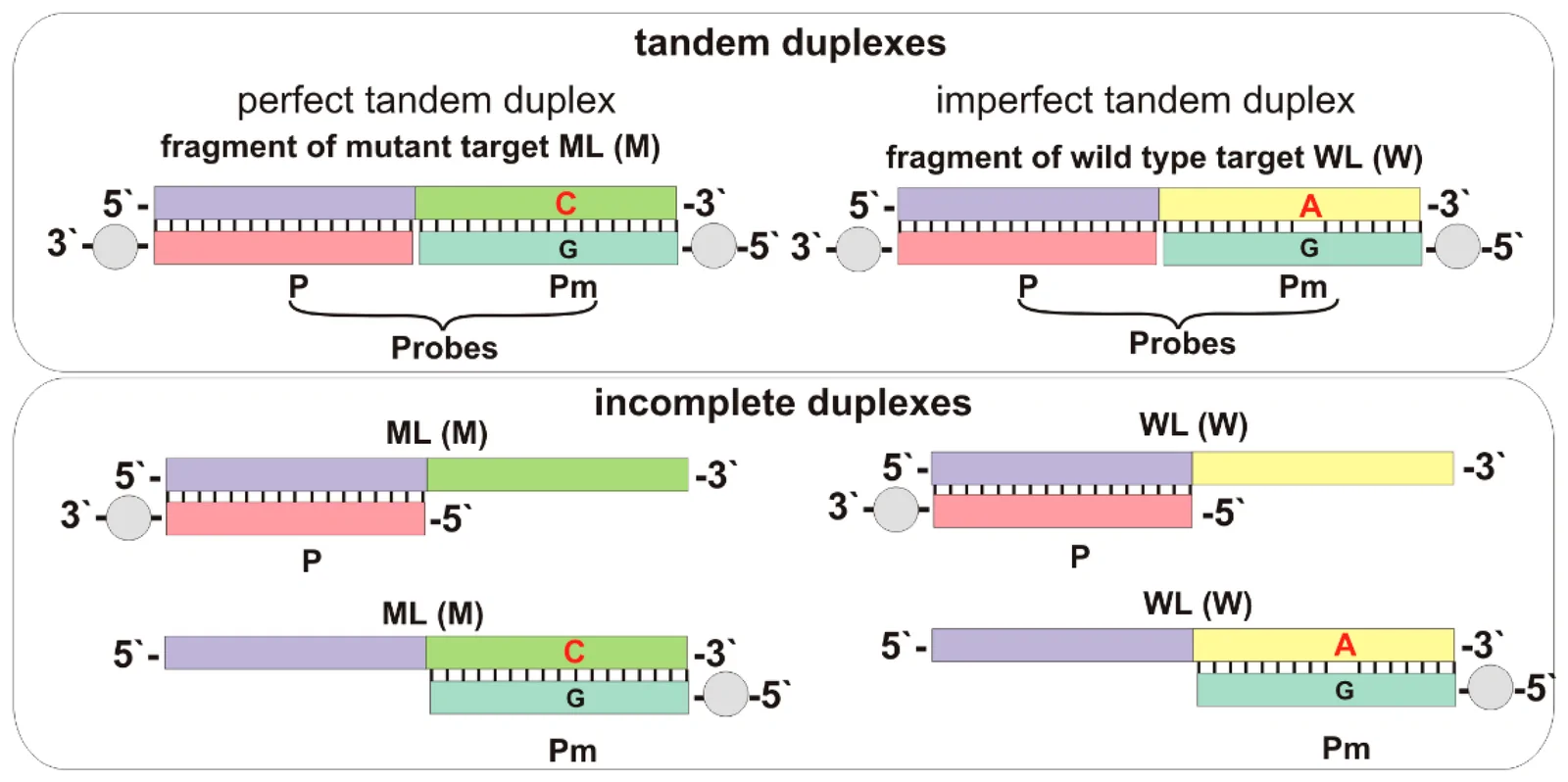
Duplexes formed by probes and targets for the detection of a single-nucleotide mutation. The gray circle denotes the functional group in the oligonucleotide probe for efficient adsorption onto AuNPs or MAuNPs. The abbreviations of W and M denote short (34 nt) wild-type and mutant DNA targets, WL and ML denote the corresponding extended (198 bp) DNA targets.

**Figure 4:**
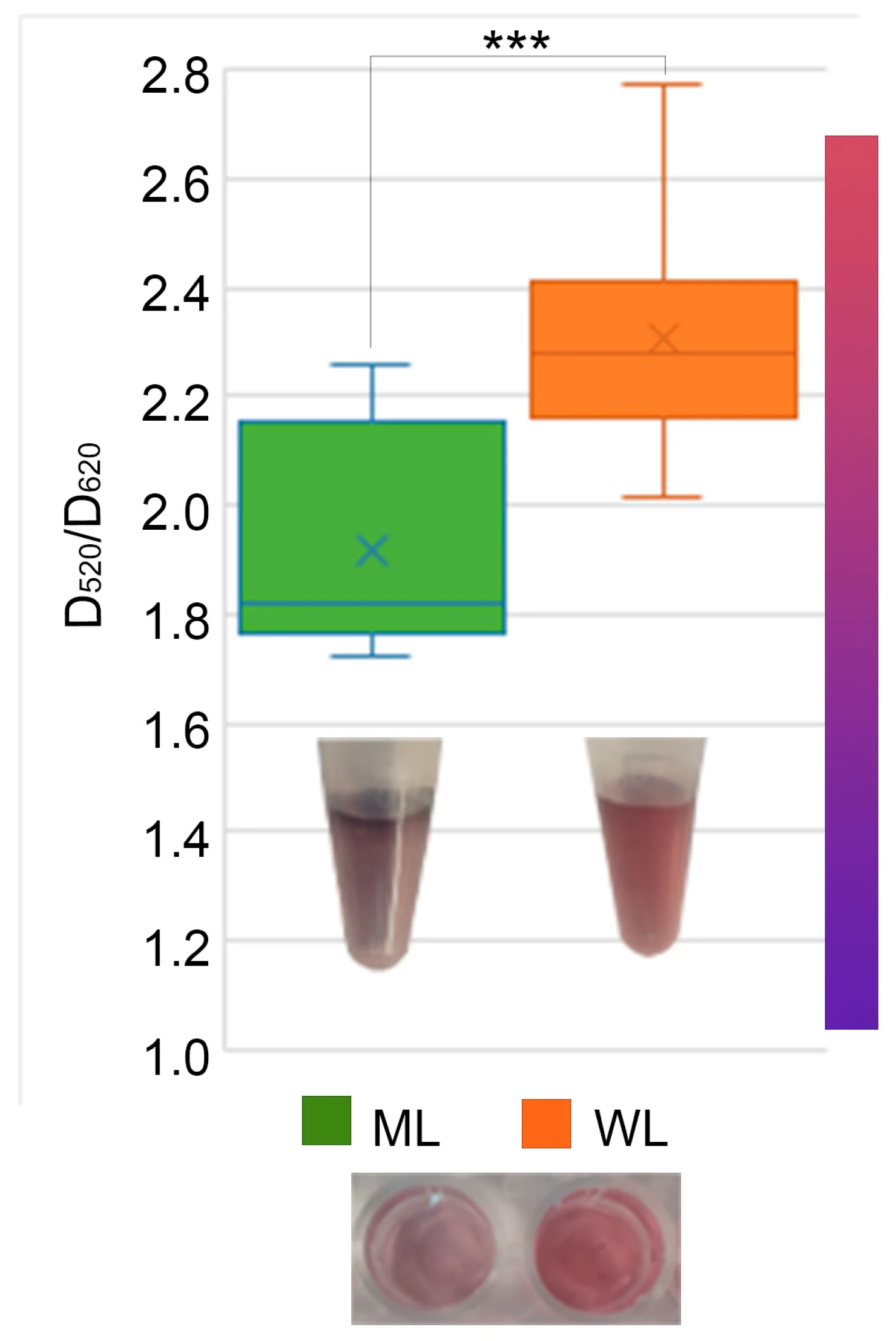
SNP detection in the mutant target (ML) and wild-type target (WL) by means of probes P and Pm adsorbed on AuNPs. Box plot of optical density at 520 nm to optical density at 620 nm (D_520_/D_620_) ratios, and photos of samples in test tubes and in plate wells are presented. The colors of the gradient panel on the right match the colors of real samples. Significant differences were found between targets: ***—*p* < 0.001.

**Figure 5:**
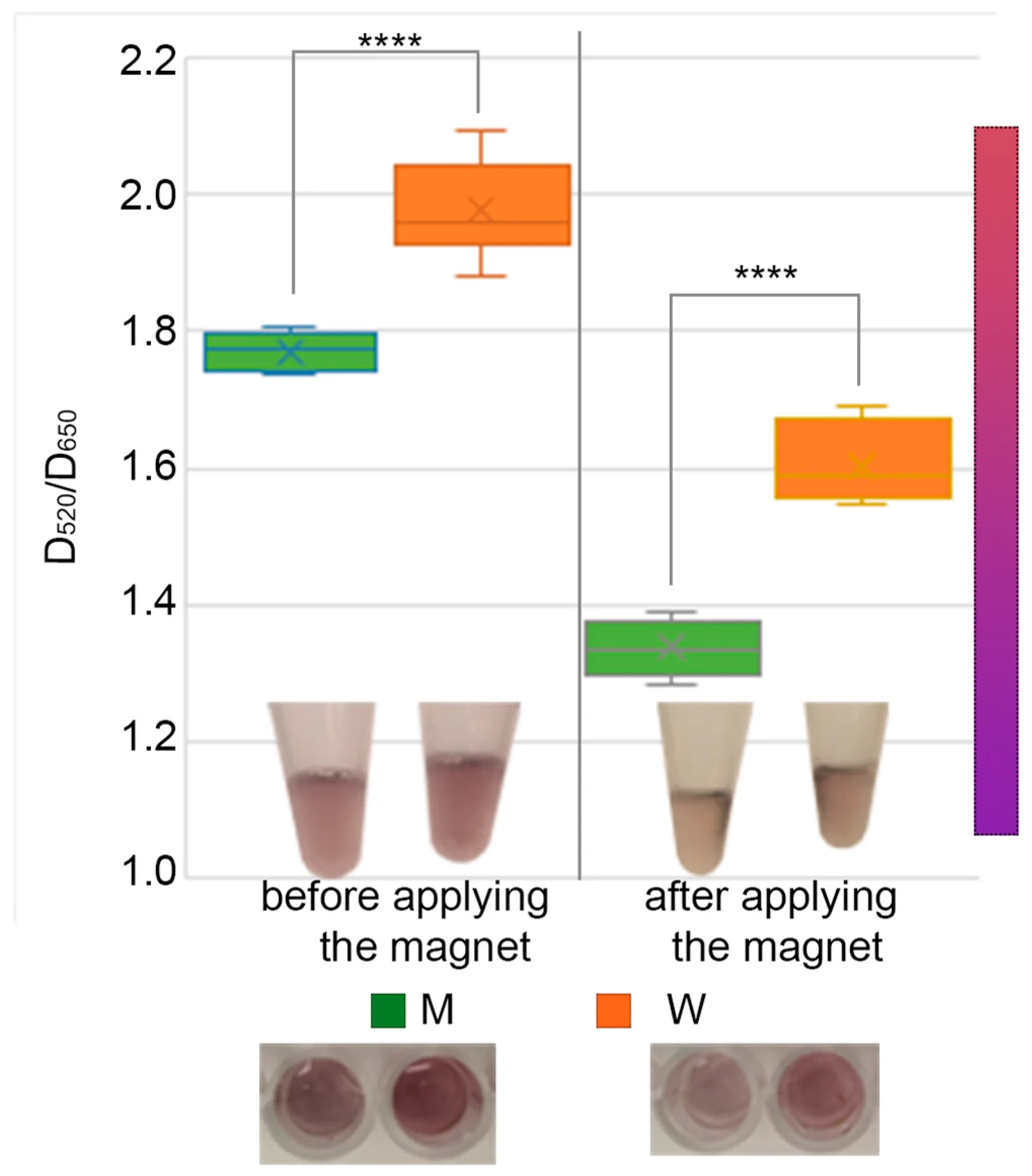
SNP detection in the mutant target (M) and wild-type target (W) by means of probes P and Pm adsorbed on AuNPs and MAuNPs. Box plot of optical density at 520 nm to optical density at 650 nm (D_520_/D_650_) ratios, photos of samples in test tubes and in wells of the plate are presented. The colors of the gradient panel on the right match the color of real samples. Enhancement of the difference between M and W targets as a result of applying an external magnetic field. Significant differences were found between targets: ****—*p* < 0.0001.

**Figure 6:**
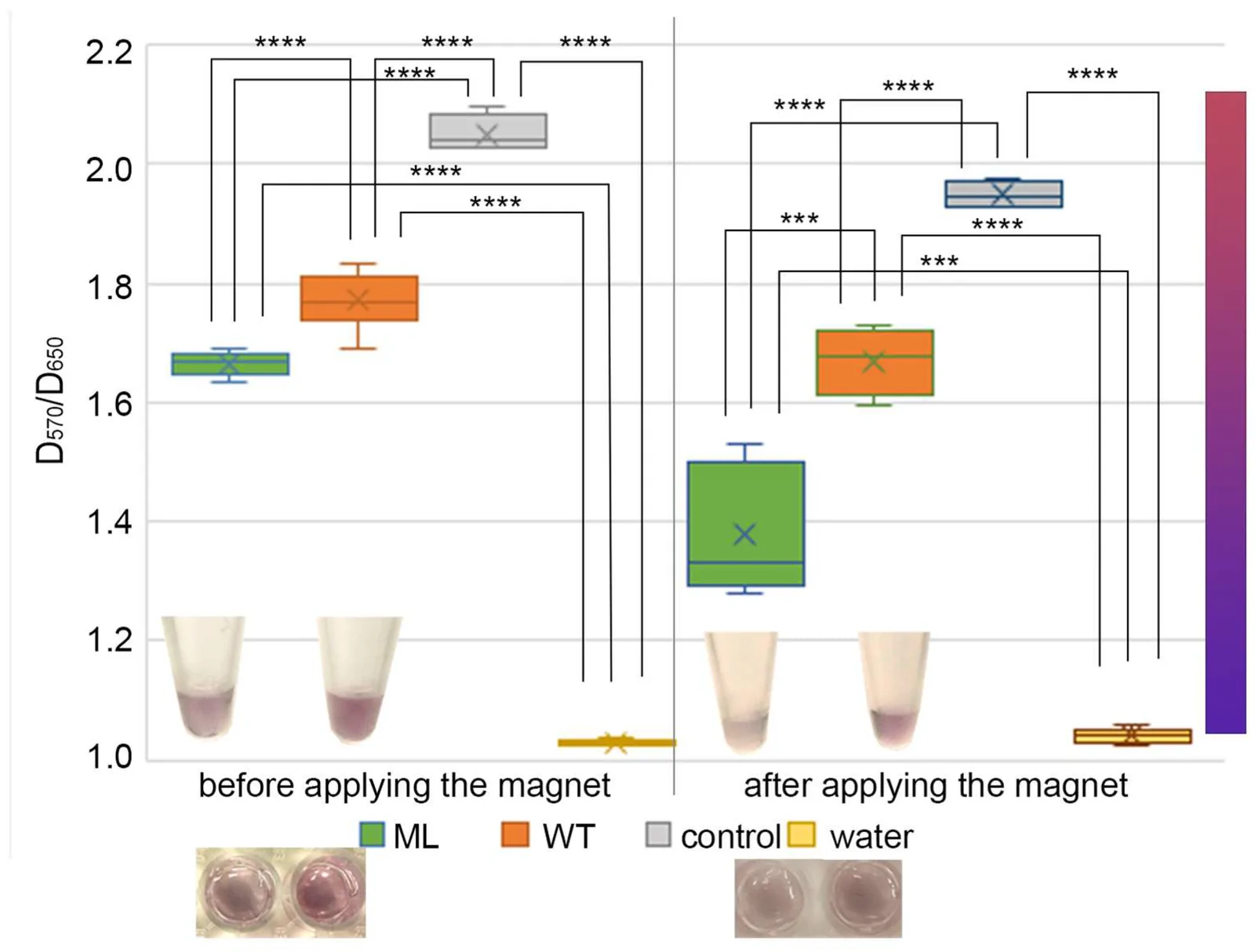
SNP detection in the mutant target (ML) and wild-type target (WL) by means of probes P and Pm conjugated with BSA and adsorbed on AuNPs and MAuNPs. Discrimination between targets differing by one nucleotide (targets ML and WL) or by sequence (targets ML or WL and control) and without any DNA target (water). Box plot of optical density at 570 nm to optical density at 650 nm (D_570_/D_650_) ratios, photos of samples in test tubes and in wells of the plate are presented. The colors of the gradient panel on the right match the color of real samples. Enhancement of the difference between M and W targets as a result of applying an external magnetic field. Significant differences were found between targets: ***—*p* < 0.001; ****—*p* < 0.0001.

**Figure 7:**
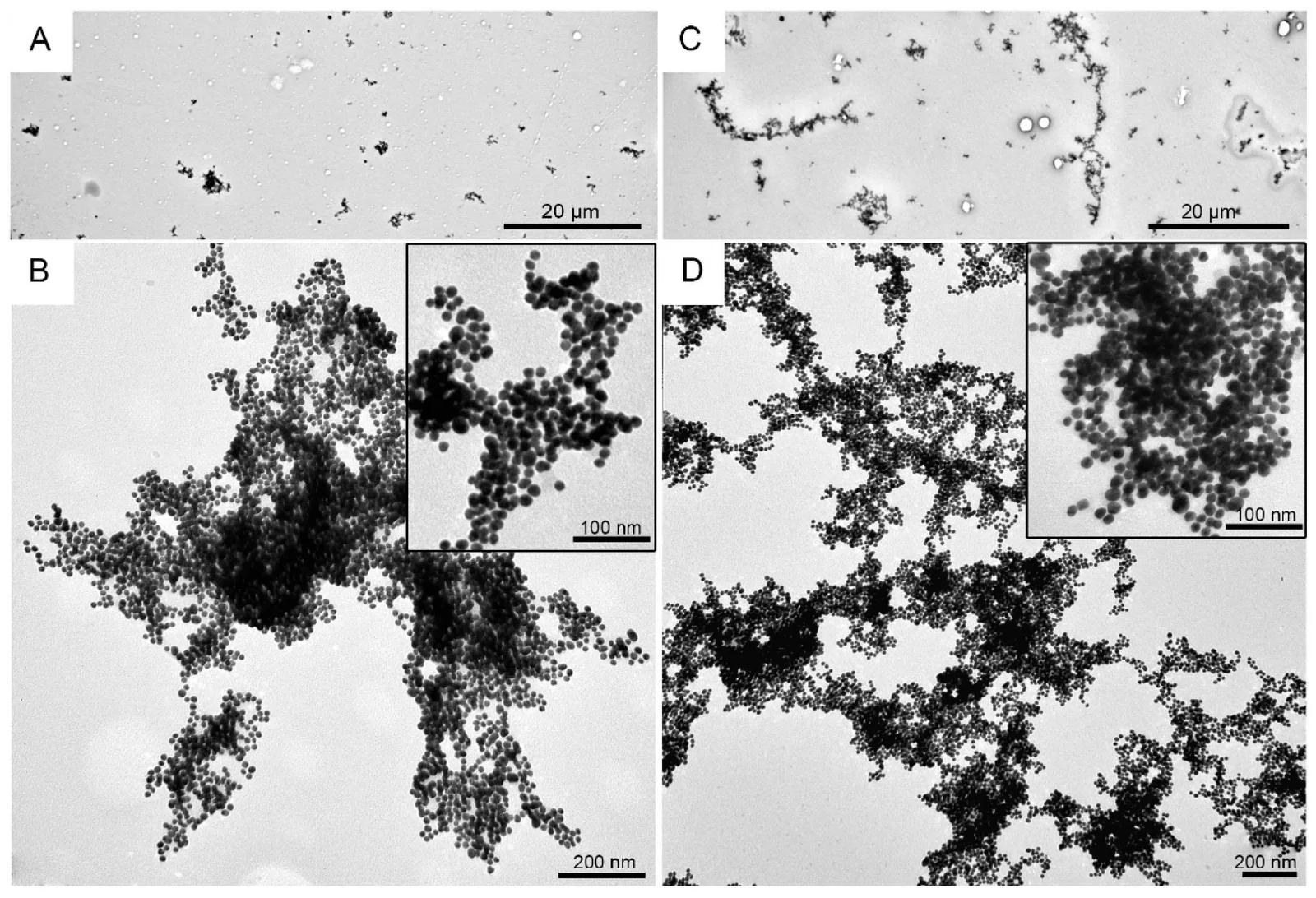
TEM (transmission electron microscopy) micrographs of supernatants after applying an external magnet to the samples featuring the probes Pm and P conjugated with BSA and two different targets: mutant (ML) (**A**,**B**) or wild-type (WL) (**C**,**D**). The scale bars are 20 μm (**A**,**C**), 200 nm (**B**,**D**), and 100 nm (insets). A measure of 2 μL of the particle suspension was applied to a 200-mesh copper grid coated with formvar film, then the grids were air-dried.

**Table 1:** Sequences of single-stranded oligonucleotides (ssDNAs).

Name	Nucleotide Sequence 5′ → 3′
F1	GGG-TGC-TCT-ATG-CAA-TGT-TCG-ATT-CCG-GCT-TCC-GCC-CGG-ACC-GCA-GCC-ACG-C
F2	p-CAA-GTC-GGC-CCG-GTC-GGT-TGC-CGA-GAC-CAT-GGG-CAA-CTA-CCA-CCC-GCA-CG
FW3	p-GCG-ACG-CGT-CGA-TCT-ACG-ACA-CCC-TGG-TGC-GCA-TGG-CCC-AGC-CCT-GGT-C
F4	p-GCT-GCG-CTA-CCC-GCT-GGT-GGA-CGG-CCA-GGG-CAA-CTT-CGG-CTC-GCC-AG
FM3	p-GCG-ACG-CGT-CGA-TCT-ACG-CCA-CCC-TGG-TGC-GCA-TGG-CCC-AGC-CCT-GGT-C
R5	p-GAA-GCC-GGA-ATC-GAA-CAT-TGC-ATA-GAG-CAC-CC
R4	p-TCG-GCA-ACC-GAC-CGG-GCC-GAC-TTG-GCG-TGG-CTG-CGG-TCC-GGG-CG
RW3	p-CAG-GGT-GTC-GTA-GAT-CGA-CGC-GTC-GCC-GTG-CGG-GTG-GTA-GTT-GCC-CAT-GGT-C
R2	p-CGT-CCA-CCA-GCG-GGT-AGC-GCA-GCG-ACC-AGG-GCT-GGG-CCA-TGC-GCA-C
R1	CTG-GCG-AGC-CGA-AGT-TGC-CCT-GGC
RM3	p-CAG-GGT-GGC-GTA-GAT-CGA-CGC-GTC-GCC-GTG-CGG-GTG-GTA-GTT-GCC-CAT-GGT-C
M	CCC-GCA-CGG-CGA-CGC-GTC-GAT-CTA-CGC-CAC-CCT-G
W	CCC-GCA-CGG-CGA-CGC-GTC-GAT-CTA-CGA-CAC-CCT-G
NH_2_-L_18_-Pm	NH_2_-L_18_-CAG-GGT-GGC-GTA-GAT-CG
P-L_18_-NH_2_	CGT-CGC-CGT-GCG-GG-L_18_-NH_2_
LipoPm	Lipo-Tr-CAG-GGT-GGC-GTA-GAT-CG
P-NH_2_	ACG-CGT-CGC-CGT-GCG-GG-NH_2_

## Data Availability

Data is available on request from the corresponding author.

## Supplementary Materials

The following supporting information can be downloaded at: https://www.mdpi.com/article/10.3390/ijms27198809/s1.

## Abbreviations

The following abbreviations are used in this manuscript:

AuNPI	gold nanoparticle
DNA/AuNP associate	nanoconstruct obtained by noncovalent adsorption of DNA and AuNP
ζ	surface potential
DLS	dynamic light scattering
TEM	transmission electron microscopy
SNP	single-nucleotide polymorphism
XRD	X-ray diffraction analysis
EDS	energy-dispersive X-ray spectroscopy
FTIR	Fourier-transform infrared spectroscopy
CD	circular dichroism
LOD	limit of detection
