# Supplementary material for: A Colorimetric Approach to Identifying Single-Nucleotide Mutations Using Gold-Based Magnetic Nanoparticles
Source: Int J Mol Sci. 2026 Oct 1;27(19):8809. doi: 10.3390/ijms27198809 (PMC13665408; doi:10.3390/ijms27198809)
Supplement: Supplementary file 1 [file ijms-27-08809-s001.zip › ijms-4565633-supplementary.pdf]

Calculation of MAuNPs yield after synthesis

The MAuNPs synthesis reaction involved 15 mg of $HAuCl_4 \cdot 4H_2O$, which corresponds to 7.2 mg of Au. The resulting yield was 21 ± 4 mg of MAuNPs. The elemental composition of the synthesized MAuNPs was analyzed by energy-dispersive X-ray spectroscopy (EDS). The Fe content was 32.44 ± 2.81 wt. %, and the Au content was 33.16 ± 3.32 wt.%. Accordingly, the final MAuNPs sample contained 7.1 ± 1.4 mg of Au, which fully corresponds to the amount of Au used in the reaction. This experiment was performed four times. These data indicate the completeness of $AuCl_4^-$ reduction to Au^0 within the MAuNPs.

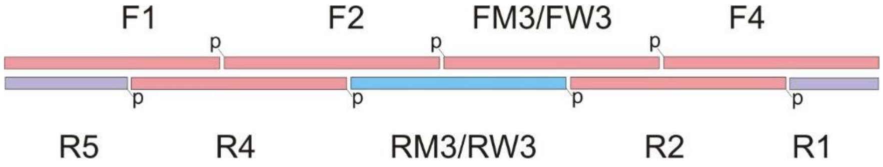

Figure S1. Scheme for obtaining extended DNA targets by the method of matrix ligation of oligonucleotides of the F* and R* series.

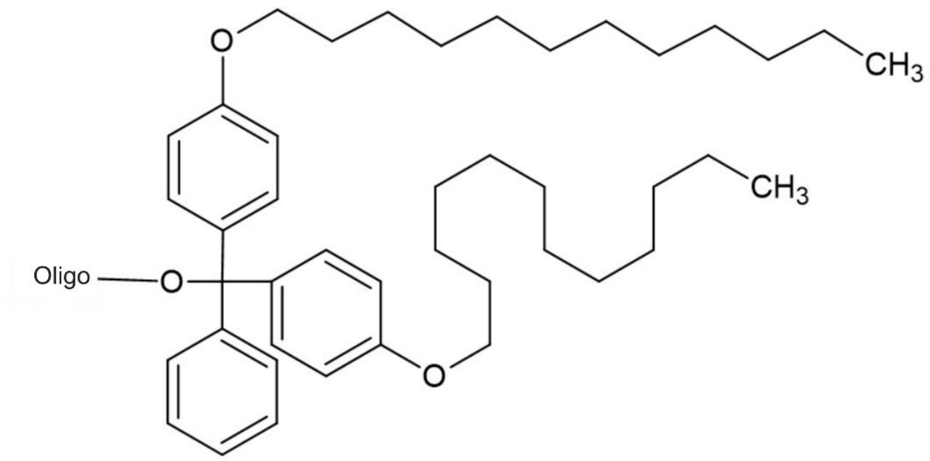

Figure S2. Structure of the Lipo residue in the oligonucleotide LipoPm.

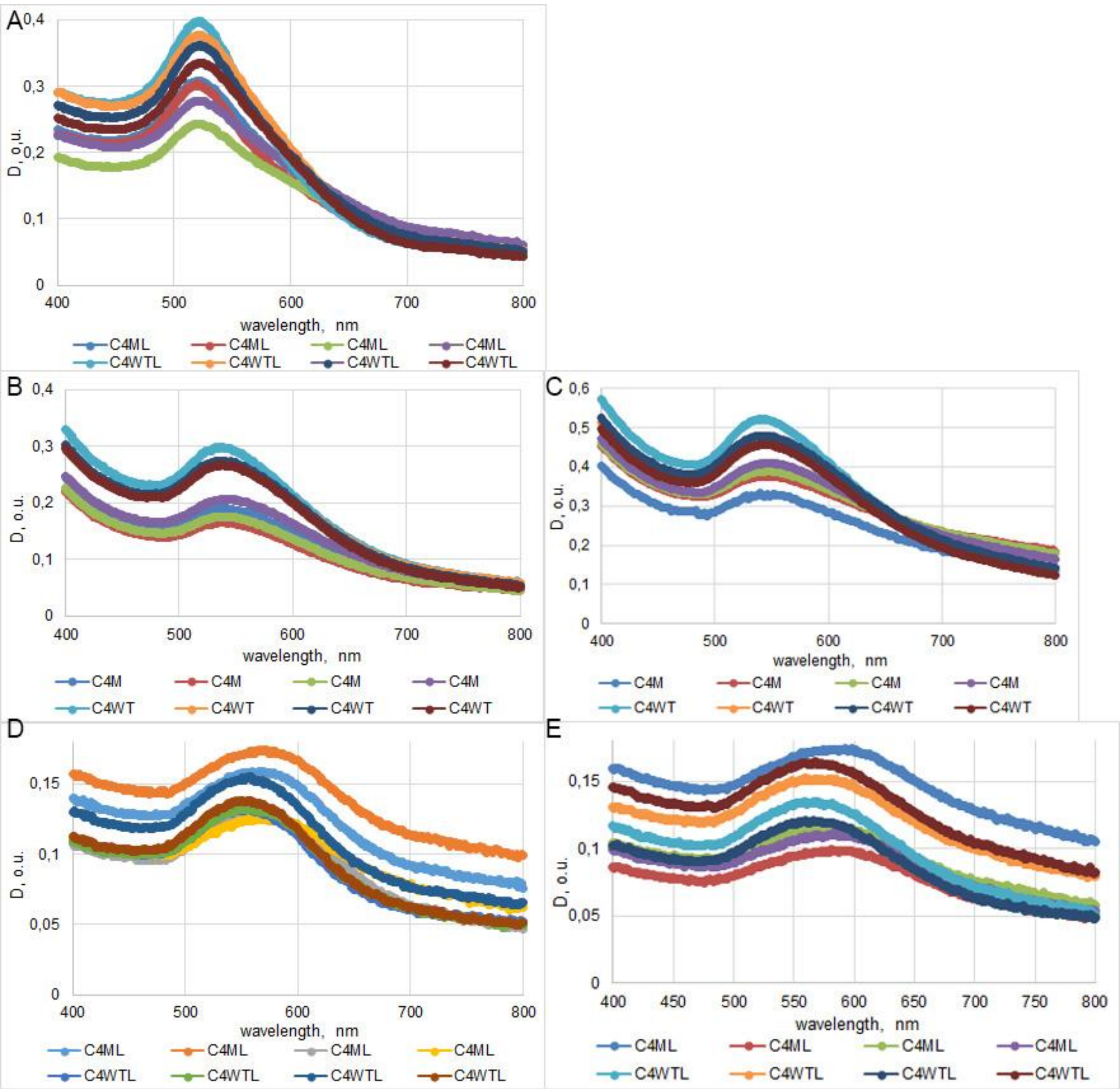

Figure S3. Typical optical absorption spectra of samples for SNP detection in extended DNA targets using AuNPs (A); in short DNA targets using AuNPs and MAuNPs before (B) and after applying an external magnetic field (C); in extended DNA targets using AuNPs and MAuNPs before (D) and after applying an external magnetic field (E).

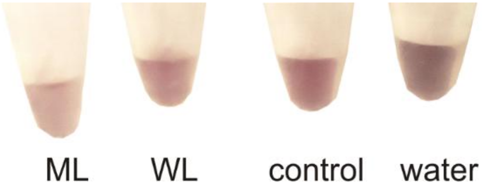

Figure S4. Photos of samples in test tubes containing targets differing by one nucleotide (targets ML and WL) or by the sequence (control target) and without any DNA target (water).

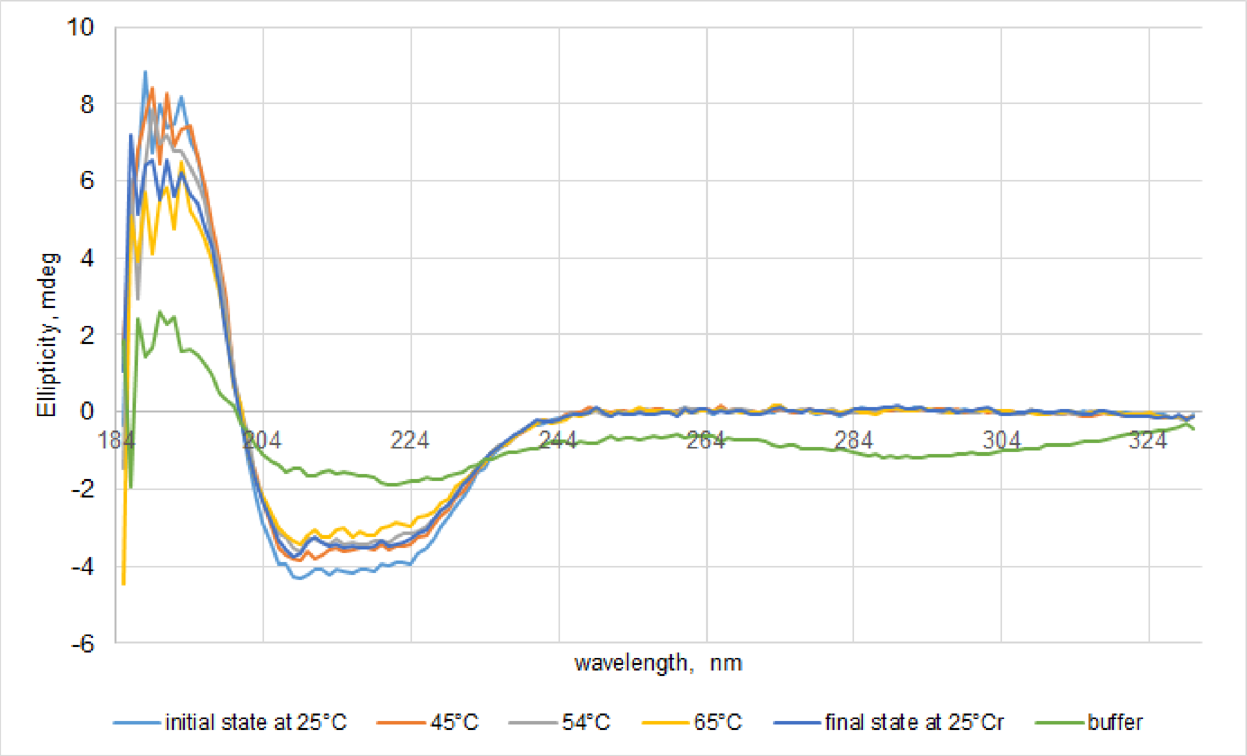

Figure S5. CD spectra of the BSA-Pm conjugate in 4.5 mM before heating, after heating to 45 °C, 54 °C, 65 °C and after cooling to 25 °C. CD spectrum for PB buffer pH 7.2 was recorded at 25 °C.
